# The effect of polyphenols on cytokine and granulocyte response to resistance exercise

**DOI:** 10.14814/phy2.13058

**Published:** 2016-12-30

**Authors:** Adam R. Jajtner, Jay R. Hoffman, Jeremy R. Townsend, Kyle S. Beyer, Alyssa N. Varanoske, David D. Church, Leonardo P. Oliveira, Kelli A. Herrlinger, Shlomit Radom‐Aizik, David H. Fukuda, Jeffrey R. Stout

**Affiliations:** ^1^ Institute of Exercise Physiology and WellnessUniversity of Central FloridaOrlandoFlorida; ^2^ Department of Exercise PhysiologyKent State UniversityKentOhio; ^3^ Exercise and Nutrition ScienceLipscomb UniversityNashvilleTennessee; ^4^ Kemin Foods L.C.Des MoinesIowa; ^5^ Pediatric Exercise and Genomics Research Center (PERC)University of California–IrvineIrvineCalifornia

**Keywords:** CD11b/CD18, exercise immunology, granulocyte colony stimulating factor, granulocyte–macrophage colony stimulating factor, inflammation, interleukin‐8

## Abstract

This study examined the effect of resistance exercise on the production, recruitment, percentage, and adhesion characteristics of granulocytes with and without polyphenol (PPB) supplementation. Thirty‐eight untrained men were randomized into three groups: PPB (*n* = 13, 21.8 ± 2.5 years, 171.2 ± 5.5 cm, 71.2 ± 8.2 kg), placebo (PL;* n* = 15, 21.6 ± 2.5 years, 176.5 ± 4.9 cm, 84.0 ± 15.7 kg), or control (CON;* n* = 10, 23.3 ± 4.3 years, 173.7 ± 12.6 cm, 77.3 ± 16.3 kg). Blood samples were obtained pre (PRE), immediately (IP), 1 h (1H), 5 h (5H), 24 h (24H), 48 h (48H), and 96 h (96H) postresistance exercise (PPB/PL) or rest (CON). Fine‐needle biopsies were obtained from the vastus lateralis at PRE, 1H, 5H, and 48H. Plasma concentrations and intramuscular content of interleukin‐8 (IL‐8), granulocyte (G‐CSF), and granulocyte–macrophage colony stimulating factor (GM‐CSF) were analyzed via multiplex assays. Changes in relative number of circulating granulocytes and adhesion receptor (CD11b) were assessed using flow cytometry. Intramuscular IL‐8 was significantly elevated at 1H, 5H, and 48H (*P *<* *0.001). Area under the curve analysis indicated a greater intramuscular IL‐8 content in PL than PPB (*P *=* *0.011). Across groups, circulating G‐CSF was elevated from PRE at IP (*P *<* *0.001), 1H (*P *=* *0.011), and 5H (*P *=* *0.025), while GM‐CSF was elevated at IP (*P *<* *0.001) and 1H (*P *=* *0.007). Relative number of granulocytes was elevated at 1H (*P *<* *0.001), 5H (*P *<* *0.001), and 24H (*P *=* *0.005, *P *=* *0.006) in PPB and PL, respectively. Across groups, granulocyte CD11b expression was upregulated from PRE to IP (*P *<* *0.001) and 1H (*P *=* *0.015). Results indicated an increase in circulating CD11b on granulocytes, and IL‐8 within the muscle following intense resistance exercise. Polyphenol supplementation may attenuate the IL‐8 response, however, did not affect granulocyte percentage and adhesion molecule expression in peripheral blood following resistance exercise.

## Introduction

Resistance exercise performed at a sufficient intensity will result in microtrauma to skeletal muscle, which may be reflected by leakage of various biomarkers (e.g., creatine kinase, CK) and/or myoglobin), increases in muscle soreness, and potential decreases in muscle performance (Clarkson and Hubal 2002; Paulsen et al. 2005). The mechanical stress associated with a resistance exercise stimulus and the resulting tissue damage signals a profound nonspecific immune response (Tidball and Villalta 2010; Freidenreich and Volek 2012). This response manifests itself through increases in cytokine and chemokine production from skeletal muscle tissue, endothelial cells, resident macrophages, and other circulating immune cells (Nieman et al. 2004; Della Gatta et al. 2014). Once released, cytokines and chemokines will elicit a response from the immune system, resulting in an accumulation of myeloid cells within a few hours, which persist for several days (Paulsen et al. 2010).

The infiltration of damaged tissue consists of three phases: preliminary, early, and late, with each phase eliciting specific actions within the recovery process (Tidball and Villalta 2010). The preliminary phase promotes an inflammatory environment (Nguyen and Tidball 2003; Pizza et al. 2005) primarily consisting of neutrophils, the most abundant granulocyte (Parkin and Cohen 2001). Granulocytes, which include neutrophils, eosinophils, and basophils, are produced within the bone marrow as a result of stimulation by granulocyte colony stimulating factor (G‐CSF) (Roberts 2005), while granulocyte–macrophage colony stimulating factor (GM‐CSF) and interleukin‐8 (IL‐8) function to activate and recruit granulocytes to the site of tissue damage (Hammond et al. 1995; Francisco‐Cruz et al. 2014). Following the preliminary phase, the early and late phases are characterized by macrophages that promote inflammation (M1) and recovery (M2), respectively (Tidball and Villalta 2010).

Changes in neutrophil counts (Peake et al. 2005b) and cellular activation (Pizza et al. 1996; Saxton et al. 2003) are observed following exercise. Macrophage‐1 antigen (MAC1), also referred to as complement receptor 3 (CR3) is a *β*2 integrin composed of CD11b and CD18, and facilitates the late phases of transendothelial migration of immune cells following tissue damage (Tan 2012). Investigations examining the expression of CD11b/CD18 on neutrophils in response to exercise have utilized various modes of exercise (Pizza et al. 1996; van Eeden et al. 1999; Saxton et al. 2003) and have yielded equivocal results (Pizza et al. 1996; Saxton et al. 2003; Peake et al. 2005b). To our knowledge, no investigations have examined the neutrophil CD11b/CD18 response following a dynamic resistance exercise bout.

As resistance exercise appears to elicit significant elevations in markers of oxidative stress (Merry and Ristow 2015), antioxidant supplementation has been examined as a potential countermeasure to reduce the oxidative response to resistance exercise (Panza et al. 2008; Jowko et al. 2011; Paulsen et al. 2014). Specifically, polyphenol supplementation has been demonstrated to reduce force deficits and markers of muscle damage in response to resistance exercise (Panza et al. 2008; Jowko et al. 2011), while others have demonstrated equivocal results (Paulsen et al. 2014). Although supplementation with antioxidants (vitamins A, C, and E) appear to blunt the response of the proinflammatory cytokines following endurance exercise (Vassilakopoulos et al. 2003), the benefits of polyphenol supplementation in conjunction with eccentric exercise have been ambiguous (Kerksick et al. 2010; O'Fallon et al. 2012; Herrlinger et al. 2015). Decreases in circulating neutrophil counts have been observed following eccentric exercise in conjunction with epigallocatechin gallate (EGCG) supplementation (Kerksick et al. 2010), though the fate of these cells is unknown. As polyphenol incubation results in reduced expression of adhesion molecules on neutrophils, along with limited chemotaxis in vitro (Kawai et al. 2004; Takano et al. 2004), decreased neutrophil concentrations following exercise are not likely explained by increased infiltration. To the best of our knowledge, the specific response of neutrophil activation following polyphenol supplementation and resistance exercise is not well understood.

Therefore, the aim of this investigation was to examine the postexercise responses of IL‐8, G‐CSF, and GM‐CSF within circulation and skeletal muscle tissue, as well as the changes in granulocyte percentage and activation (CD11b/CD18) following an acute bout of resistance exercise designed to elicit muscle damage. Furthermore, we examined the influence of a proprietary polyphenol supplement on this response following an acute bout of resistance exercise.

## Methods

### Participants

Thirty‐eight healthy recreationally active, but untrained, men (18–35 years old) volunteered to participate in this study. Participants were randomly assigned to one of three groups. The first group consumed 2 g per day of a proprietary polyphenol blend (PPB) supplement, the second group consumed 2 g per day of a placebo (PL), and the third group served as control (CON), with no supplement or exercise. The anthropometric performance and compliance characteristics of the 38 participants are displayed in Table 1. Following an explanation of all procedures, risks, and benefits, each participant provided informed written consent prior to completing any testing. For inclusion in the study, participants had to participate in <3 h of planned exercise per week, have a body mass index of 18.0–34.9 kg/m^2^, be free of physical limitations, and be willing to maintain a habitual diet while abstaining from tea, alcohol, and additional dietary supplements.

**Table 1:** Participant baseline characteristics

	Polyphenol blend (PPB)	Placebo (PL)	Control (CON)	*P*
*n*	13	15	10	N/A
Age (years*)*	21.8 ± 2.5	21.6 ± 2.5	23.3 ± 4.3	0.367
Height (cm)	171.2 ± 5.5	176.5 ± 4.9	173.7 ± 12.6	0.213
Body mass (kg)	71.2 ± 8.2	84.0 ± 15.7	77.3 ± 16.3	0.063
BMI (kg*m^−1^)	24.3 ± 2.8	26.9 ± 4.2	25.4 ± 3.4	0.182
Squat 1‐RM (kg)	108.2 ± 14.4	108.6 ± 30.5	119.8 ± 31.5	0.514
Leg press 1‐RM (kg)	158.0 ± 41.4	160.6 ± 30.5	192.5 ± 74.6	0.315
Compliance (%)	95.9 ± 6.6	95.4 ± 6.5	N/A	0.853
Calories (kcal)	2038 ± 473	2171 ± 556	2027 ± 601	0.777
Carbohydrates (g)	234 ± 56	258 ± 82	238 ± 81	0.944
Protein (g)	97 ± 18	101 ± 31	100 ± 41	0.685
Fat (g)	82 ± 23	83 ± 32	77 ± 27	0.856

Data are presented as mean ± SD.

### Study design

**Figure 1:**
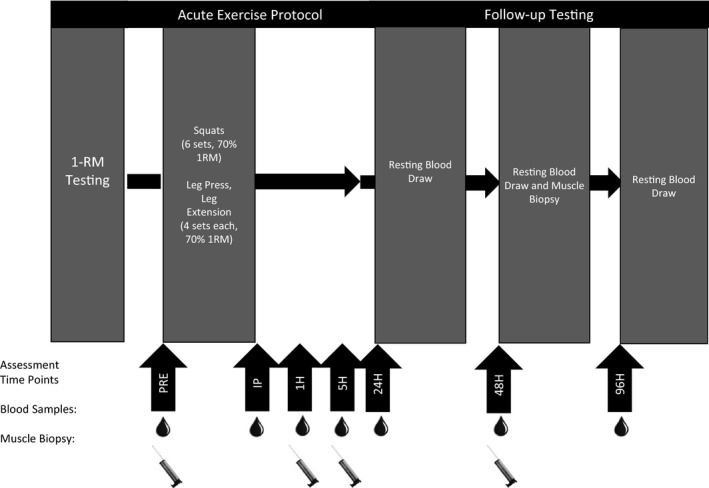
Study design. Participants completed 1‐RM testing at least 72 h prior to the exercise protocol. During day 2, participants completed a muscle damaging workout and provided blood samples pre (PRE), immediately (IP), 1 h (1H), and 5 h (5H) post exercise. Participants provided additional blood samples 24 h (24H), 48 h (48H), and 96 h (96H) following damage. Skeletal muscle biopsies were obtained PRE, 1H, 5H, and 48H.

For this randomized, placebo‐controlled trial, all participants reported to the Human Performance Lab for 5 days of testing (Fig. 1). Prior to the first day of testing, PPB and PL completed a 28‐day supplementation protocol. Day 1 consisted of one‐repetition maximum (1‐RM) testing of squat, leg press, and leg extension exercises, and occurred at least 72 h prior to the second day of the study. On day 2, participants arrived in the laboratory after fasting for 12 h, and provided a resting blood sample and muscle biopsy (PRE). After blood samples were obtained, participants were provided a small breakfast bar (cal: 190, CHO: 19 g, protein: 7 g, fat: 13 g). After participants consumed their breakfast bar, CON rested for 1 h, while PPB and PL began the acute, resistance exercise protocol. Participants provided blood samples immediately (IP), 1 h (1H), and 5 h (5H) postexercise (PPB and PL) or 1 h rest (CON). Additionally, muscle biopsies were obtained at 1H and 5H postexercise. Following the 1H muscle biopsy, participants were provided with a light meal (cal: 250, CHO: 34 g, protein: 14 g, fat: 6 g). Participants returned to the laboratory in a fasted state 24 h (24H), 48 h (48H), and 96 h (96H) later for resting blood samples, and an additional muscle biopsy at 48H. Adherence to the fasting and diet criteria (no tea, alcohol, or additional supplements) was verbally confirmed each morning with participants as they arrived for testing.

### Supplementation protocol

Both the PPB and PL groups completed daily supplementation for 28 days. The PPB group consumed a proprietary polyphenol blend of water‐extracted green and black tea (*Camellia sinensis*) containing minimum 40% total polyphenols, 1.3% theaflavins, 5–8% epigallocatechin 3‐gallate (EGCG), 7–13% caffeine, and 600 ppm manganese (Kemin Foods, L.C., Des Moines, IA). The PL group consumed microcrystalline cellulose in capsules of similar shape, color, and size. All products were tested for toxins including heavy metals, pesticides, and excipients by an independent third party.

Briefly, participants reported to the Human Performance Lab 3–5 days per week to receive the supplement. Participants took one dose (1 g PPB or PL) under the supervision of a member of the research team, and were given their prescribed doses in individual containers (1 g PPB or PL) for each additional time point. Participants consumed two 1000 mg doses per day for a total of 2000 mg per day of either PPB or PL daily. Participants were asked to return all empty containers upon their next visit to the laboratory. Participants who did not maintain 80% compliance in each phase (28 days of supplementation or during the exercise protocol and recovery) were removed from analysis.

### Nutritional analysis

Participants were provided a food log for the 2 days prior testing, as well as each day of the testing protocol (for a total of 6 days). Each day participants reported to the laboratory and members of the research team reviewed the logs with each participant. The USDA Nutritional Database (U.S. Department of Agriculture, Beltsville, MD) was used to analyze total calories, carbohydrates, protein, and fat. Values are represented as the average intake across the 6 days of data collection.

### One‐repetition maximum testing

Direct measurement of 1‐RM maximal strength was completed on the squat and leg press exercises, while a predicted 1‐RM was performed on the leg extension exercise. All participants completed a standardized warm‐up, consisting of 5 min on a cycle ergometer against a self‐selected resistance, 10 body weight squats, 10 walking lunges, 10 dynamic hamstring stretches, and 10 dynamic quadriceps stretches. All 1‐RM testing was completed as described previously (Hoffman 2006). Briefly, each participant completed two warm‐up sets consisting of 5–10 repetitions and 3–5 repetitions at approximately 40–60% and 60–80% of his perceived maximum, respectively. Each participant then performed up to five subsequent trials to determine his 1‐RM with 3–5 min of rest between each set. Exercise order was kept consistent between participants, with 3–5 min of rest between the squat, leg press, and leg extension.

During the squat exercise, participants placed a safety squat bar (Power Lift, Jefferson, IA) across their shoulders and descended to the parallel position, where the greater trochanter of the femur reached the same level as the knee. Participants then ascended to a complete knee extension. Leg press was completed with the participant sitting in a reclined position, with their legs extended. Participants were asked to lower the weight until the lower leg and femur created a 90° angle. Participants were then asked to press the weight up. Participants who were unable to complete the repetition or maintain proper range of motion were given one additional opportunity. If they were still unable to perform the exercise correctly, the last completed weight was recorded as the 1‐RM.

For the leg extension exercise, participants were placed in a seated position and were asked to extend their legs to 180° at the knee or full extension. Participants were asked to perform as many repetitions as possible, and the resulting repetitions and weight used were applied to a prediction equation (Brzycki 1993). If more than 10 repetitions were performed, the weight was increased and the participant repeated the measure 3–5 min later. All testing was observed by a certified strength and conditioning specialist to monitor adherence to form.

### Acute exercise protocol

Only PPB and PL completed the acute resistance exercise protocol, while CON rested for an hour. The exercise protocol designed to cause muscle damage in previously untrained individuals was preceded by a light warm‐up as described above. Following the light warm‐up, participants completed a resistance exercise session that consisted of six sets of 10 repetitions of the squat as well as four sets of 10 repetitions of the leg press and leg extension exercises. All exercises were completed at 70% of the subjects’ previously determined 1‐RM with 90 sec of rest between each set. Participants were provided with assistance if they were unable to complete 10 repetitions on their own, and weight for the subsequent set was reduced. All testing sessions were observed by a certified strength and conditioning specialist to monitor adherence to exercise form.

### Blood sampling

Blood samples were obtained at seven time points throughout the study (PRE, IP, 1H, 5H, 24H, 48H, and 96H). The PRE, IP, and 1H blood samples were obtained using a Teflon cannula placed in a superficial forearm vein using a three‐way stopcock with a male Luer‐lock adapter and plastic syringe. The cannula was maintained patent using an isotonic saline solution (Becton Dickinson, Franklin Lakes, NJ). PRE and 1H blood samples were obtained following a 15‐min equilibration period, while IP blood samples were taken within 5 min of exercise cessation. The remaining time points (5H, 24H, 48H, and 96H) were obtained using a single use disposable needle with the subject in a supine position for at least 15 min prior to sampling. Whole blood (20 mL) was collected in two Vacutainer^®^ tubes (Becton Dickinson, Franklin Lakes, NJ), one containing K_2_‐EDTA and one containing no anticlotting agents. Aliquots were removed from the first tube for hematocrit and hemoglobin measures, as well as flow cytometry analysis, while the second tube was allowed to clot for 30 min prior to being centrifuged at 3000*g* for 15 min with the remaining whole blood from the first tube. The resulting plasma and serum was aliquoted and stored at −80°C for later analysis.

Hematocrit was analyzed in duplicate from whole blood via microcentrifugation (Statspin^®^, Critspin, Westwood, MA) and microcapillary technique. Hemoglobin was analyzed in duplicate from whole blood using an automatic analyzer (Hemocue^®^, Cypress, CA). Coefficient of variation for each assay was 0.20% for hematocrit and 0.46% for hemoglobin. Plasma volume shifts following the workout were calculated via the formula established by Dill and Costill (1974); however, circulating values were not adjusted to account for changes in plasma volume.

### Markers of muscle damage

Serum concentrations of myoglobin (MG) were obtained via enzyme‐linked immunosorbent assay (ELISA) (Calbiotech, Spring Valley, CA), while CK was analyzed using a commercially available kinetic assay (Sekisui Diagnostics, Charlottetown, PE, Canada) as per manufacturer's instructions. To limit interassay variability, all samples for a particular assay were thawed once, and analyzed by the same technician using a BioTek Eon spectrophotometer (BioTek, Winooski, VT). All samples were analyzed in duplicate with a mean coefficient of variation of 7.57% for MG and 3.66% for CK.

### Circulating cytokine concentration

Plasma concentrations of IL‐8, G‐CSF, and GM‐CSF were analyzed via multiplex assay using the human cytokine/chemokine panel one (EMD Millipore, Billerica, MA). All samples were thawed once and analyzed in duplicate by the same technician using the MagPix (EMD Millipore), with mean coefficient of variation of 8.04%, 7.82%, and 7.10% for IL‐8, G‐CSF, and GM‐CSF, respectively.

### Fine‐needle skeletal muscle biopsy procedure

Fine‐needle muscle biopsies were performed on the vastus lateralis muscle of the participant's dominant leg using a spring‐loaded, reusable instrument with 14‐gauge disposable needles and a coaxial introducer (Argon Medical Devices, Inc., Plano, TX). Following local anesthesia with 2 mL of 1% lidocaine applied into the subcutaneous tissue, a small incision to the skin was made and an insertion cannula was placed perpendicular to the muscle until the fascia was pierced (Townsend et al. 2016). The biopsy needle was inserted through the cannula and a muscle sample was obtained by the activation of a trigger button, which unloaded the spring and activated the needle to collect a muscle sample. Each muscle sample was removed from the biopsy needle using a sterile scalpel and was subsequently placed in a cryotube, rapidly frozen in liquid nitrogen, and stored at −80°C. A new incision was made for each time point, with approximately 2 cm between all sampling sites. All muscle biopsies were performed by a licensed medical physician.

### Intramuscular cytokine protein content

Sufficient sample was not obtained from nine participants (PPB = 2, PL = 3, CON = 4), and therefore were not included in the intramuscular analysis. Tissue samples were thawed and kept on ice for preparation and homogenization. A proprietary lysis buffer with protease inhibitor (EMD Millipore) was added to each sample at a rate of 500 *μ*L per 10 mg of tissue. Samples were homogenized using a Teflon pestle and sonication (Branson, Danbury, CT, USA). Tissue samples were then agitated for 10 min at 4°C and centrifuged at 10,000*g* for 5 min. The supernatant was then aspirated and used for analysis.

Total protein content was assessed using a detergent compatible (DC) protein assay kit (Bio‐Rad), and samples were diluted to 0.8–1.2 mg/mL. Protein content of the cytokines IL‐8, G‐CSF, and GM‐CSF were then assessed via multiplex assay (EMD Millipore) as per manufacturer's guidelines, and normalized to the total protein content. To limit interassay variance, all tissue samples were analyzed in duplicate in the same assay run by a single technician. Average coefficient of variation was 7.46%, 13.01%, and 5.04% for IL‐8, G‐CSF, and GM‐CSF, respectively. Intramuscular cytokine protein content is expressed as pg/*μ*g total protein (Della Gatta et al. 2014).

### Leukocyte preparation

Fresh, anticoagulated (K_2_‐EDTA), whole blood (100 *μ*L) was mixed with fluorescent‐conjugated monoclonal antibodies specific to CD11b‐fluorescein isothiocyanate (FITC; Biolegend, San Diego, CA) and CD66b‐phycoerythrin (PE; BD Biosciences, San Jose, CA). Samples were mixed and incubated for 15 min in the dark, after which the samples were lysed with 2 mL of 1× FACS lysing solution (BD Biosciences), mixed and incubated in the dark for an additional 8 min. Following incubation, samples were centrifuged at 300*g* for 8 min and washed with 2 mL of 1× wash buffer containing 1% fetal bovine serum (FBS) in a 1× phosphate‐buffered saline (PBS) solution. Samples were centrifuged again at 300*g* for 8 min, and the supernatant was removed. Samples were then fixed in 300 *μ*L of 2% paraformaldehyde in PBS.

### Flow cytometry analysis

Cell preparations were acquired using an Accuri C6 flow cytometer (BD Accuri Cytometers, Ann Arbor, MI) equipped with two lasers providing excitation at 488 and 640 nm, and four band‐pass filters (FL1: 533/30, FL2: 585/40, FL3 670LP, FL4: 675/25). Events were recorded based on size (forward scatter area; FSC‐A), complexity (side scatter area; SSC‐A), and mean fluorescence intensity (MFI). A total of 200 *μ*L were collected for each sample, which ensured at least 10,000 CD66b+ events.

**Figure 2:**
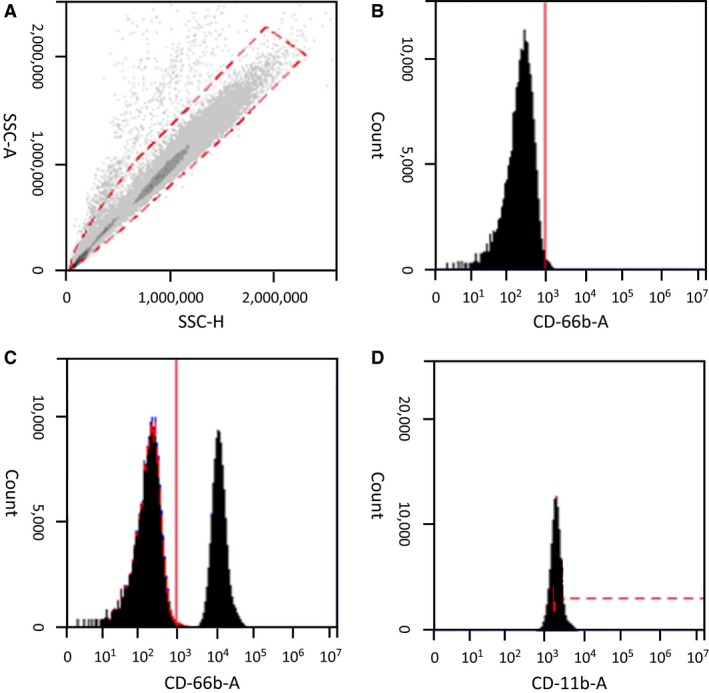
Gating procedure. All samples were initially gated for multiplet exclusion (A). Granulocytes were identified by staining for CD66b in an unstained control sample (B), and compared to samples positively stained for CD66b (C). Granulocytes were then analyzed for CD11b expression (F).

Analysis was completed using BD Accuri analysis software (BD Accuri Cytometers). Events were initially gated based on side scatter height (SSC‐H) and SSC‐A as a multiplet cell exclusion criteria (Fig. 2A). Granulocytes were then determined by CD66b+ staining compared to an unstained control (Fig. 2B and C). MFI of CD11b was then determined for CD66b+ granulocytes (Fig. 2D). CD66b+ granulocytes are expressed as a percent of total leukocytes following multiplet exclusion.

### Statistics

Changes in markers of muscle damage, circulating and intramuscular cytokines, as well as granulocyte characteristics were analyzed by a two‐way, between‐subjects repeated measures analysis of variance (ANOVA). In the event of a significant F ratio, a one‐way, within‐subjects repeated measures ANOVA for each group and a one‐way, between‐subjects ANOVA at each time point with LSD pairwise comparisons were used for post hoc analysis. Significant time and group effects were subsequently analyzed with LSD pairwise comparisons. Non‐normally distributed data were transformed using the natural log (LN). Area under the curve (AUC) was also calculated for changes in circulating cytokines and myoglobin response using a standard trapezoidal technique, and a one‐way ANOVA was used to examine differences among groups. Raw concentrations from PRE, IP, 1H, and 5H were used to calculate AUC prior to LN transformation. Additionally, Pearson's product–moment correlations were calculated to examine selected bivariate relationships between granulocytes and markers of muscle damage as well as intramuscular and circulating cytokines. Significance was accepted at an alpha level of *P* ≤ 0.05 and all data are reported as mean ± SD of the original, nontransformed data.

## Results

### Participant characteristics

Forty‐eight participants were initially recruited for this investigation, of which 10 were removed prior to analysis (PPB = 6, PL = 4) for a total of 38 participants (Table 1). Of the 10 participants removed, 5 participants requested to discontinue testing (PPB = 4, PL = 1). Of the five participants that wished to discontinue, one completed a portion of the resistance exercise protocol prior to discontinuing the study (PPB group), two reported unresolvable scheduling conflicts (both from PPB group), and two discontinued supplementation during the 28‐day supplementing period (PPB = 1, PL = 1). Five additional participants who completed testing were removed from analysis due to lack of compliance (PPB = 2, PL = 3). Four of the five participants were removed for failure to achieve 80% compliance with supplementation (PPB = 2, PL = 2), while one did not adhere to the fasting requirements (PL group). No significant differences were observed between groups for participant characteristics or compliance.

### Nutritional analysis

Nutritional intake data are presented in Table 1. No significant interactions were observed between groups for the average intake of total calories, carbohydrates, protein, or fat.

### Plasma volume shifts

A significant interaction was observed for changes in plasma volume (*F* = 9.818, *P *<* *0.001, *η* ^2^ = 0.359). Changes in plasma volume at IP were significantly less in CON (−1.5 ± 4.8%) compared to PPB (−17.7 ± 5.8%; *P *=* *0.001) and PL (−15.1 ± 4.3%; *P *=* *0.001). Furthermore, at 5H, PL (0.7 ± 3.7%) was significantly greater than PPB (−4.0 ± 7.3%; *P *=* *0.038) and CON (−4.5 ± 5.8%; *P *=* *0.047). Considering the importance of the cytokine to the receptor ratio, circulating markers were not corrected for changes in plasma volume.

### Markers of muscle damage

Changes in circulating myoglobin and CK concentrations are depicted in Table 2. A significant interaction (*F* = 17.20, *P *<* *0.001, *η* ^2^ = 0.503) was observed for changes in myoglobin concentrations. Post hoc analysis indicated a significant elevation at IP, 1H, and 5H in both PPB and PL compared to CON. No significant differences in the myoglobin AUC response were observed between the groups (*F* = 1.935, *P *=* *0.160).

**Table 2:** Circulating biomarkers

	PRE	IP	1H	5H	24H	48H	96H
Myoglobin (ng mL^−1^)							
PPB	21.2 ± 12.9	59.3 ± 29.0^a^, ^b^	128.4 ± 78.2[Link], b	129.9 ± 84.3^a^, ^b^	**—**	**—**	**—**
PL	39.6 ± 56.9	74.4 ± 78.5^a^, ^b^	129 ± 119.8^a^, ^b^	98.5 ± 66.3^a^, ^b^	**—**	**—**	**—**
CON	22.1 ± 7.7	28.7 ± 7.1^a^	26.1 ± 7.5^a^	31.8 ± 11.7^a^	**—**	**—**	**—**
Creatine kinase (U∙L^−1^)							
PPB	89.2 ± 98.5	**—**	**—**	**—**	284.8 ± 238.0^a^, ^b^, ^c^	272.1 ± 202.9^a^, ^b^, ^c^	334.7 ± 264.4^a^, ^b^, ^c^
PL	70.5 ± 65.6	**—**	**—**	**—**	158.2 ± 151.6^b^, ^a^	147.2 ± 146.6^a^	151.9 ± 150.2^a^
CON	68.0 ± 51.5	**—**	**—**	**—**	74.8 ± 46.6	77.4 ± 56.5	68.5 ± 59.3
G‐CSF (pg mL^−1^)		d	d	d			
PPB	63.2 ± 26.0	79.2 ± 40.7	77.4 ± 32.6	80.5 ± 26.1	63.8 ± 23.2	56.4 ± 21.4	58.1 ± 14.9
PL	82.8 ± 50.2	95.8 ± 60.8	83.2 ± 32.1	81.0 ± 46.9	83.7 ± 56.9	86.1 ± 70.2	75.4 ± 36.4
CON	69.3 ± 26.3	103.1 ± 66.0	96.1 ± 58.0	80.9 ± 34.8	64.1 ± 29.2	67.3 ± 36.3	62.4 ± 27.5
GM‐CSF (pg∙ml^−1^)		d	d	d			
PPB	48.6 ± 37.6	53.6 ± 28.4	55.3 ± 32.5	60.6 ± 58.1	45.7 ± 34.4	44.5 ± 35.0	37.4 ± 20.6
PL	71.4 ± 69.9	96.7 ± 137.4	92.8 ± 139.3	84.1 ± 116.1	80.9 ± 108.0	78.6 ± 92.7	67.3 ± 74.7
CON	34.3 ± 19.8	52.4 ± 32.2	43.7 ± 26.7	35.6 ± 24.7	32.9 ± 20.7	33.0 ± 24.3	26.5 ± 14.6
IL‐8 (pg∙ml^−1^)							
PPB	43.1 ± 46.1	41.2 ± 42.4	48.3 ± 55.0	50.8 ± 63.2	44.3 ± 50.9	40.9 ± 43.6	33.3 ± 28.7
PL	43.9 ± 35.0	50.8 ± 42.2	51.7 ± 51.1	51.9 ± 43.6	54.3 ± 43.7	48.8 ± 36.2	48.6 ± 36.3
CON	31.5 ± 24.9	34.3 ± 31.8	31.6 ± 29.1	35.8 ± 26.3	30.3 ± 22.8	35.7 ± 39.4	25.4 ± 19.0

Supplement (PPB), placebo (PL), and control (CON) groups were analyzed for changes in myoglobin, creatine kinase (CK), granulocyte colony stimulating factor (G‐CSF), granulocyte–macrophage colony stimulating factor (GM‐CSF), and interleukin‐8 (IL‐8) from pre‐exercise (PRE) to immediately (IP), 1 h (1H), 5 h (5H), 24 h (24H), 48 h (48H), and 96 h (96H) post exercise.

Data presented as mean ± SD.

a Significant difference from PRE (*P* < 0.05).

b Significant difference from CON (*P* < 0.05).

c Significant difference from PL (*P* < 0.05).

Significant main effect of time compared to PRE, 24H, 48H, and 96H (*P* < 0.05).

d Significant main effect of time compared to 48H and 96H (*P* < 0.05).

A significant interaction was observed (*F* = 4.203, *P *=* *0.006, *η* ^2^ = 0.203) for CK concentrations. Significant elevations from PRE were noted in both PPB and PL at 24H, 48H, and 96H, with no changes observed in CON. CK concentrations at 24H, 48H, and 96H were significantly greater in PPB compared to PL and CON, while PL was significantly greater than CON at 24H only.

### Circulating cytokines

Data from circulating cytokine concentrations are also displayed in Table 2. No significant interaction (*F* = 0.876, *P *=* *0.534, *η* ^2^ = 0.052) was observed for G‐CSF; however, a significant main effect for time was observed (*F* = 7.415, *P *<* *0.001, *η* ^2^ = 0.188). When groups were collapsed, pairwise comparisons indicated significant elevations from PRE at IP, 1H, and 5H. Additionally, IP, 1H, and 5H were significantly greater than 24H, 48H, and 96H. No significant between‐group differences were observed for the G‐CSF AUC response (*F* = 0.146, *P *=* *0.732).

No significant interactions were observed for GM‐CSF (*F* = 0.556, *P *=* *0.823, *η* ^2^ = 0.032); however, a significant main effect for time was observed (*F* = 8.660, *P *<* *0.001, *η* ^2^ = 0.203). When groups were collapsed, pairwise comparisons indicated a significant elevation from PRE at IP and 1H. GM‐CSF was also significantly greater at IP and 1H than 5H, 24H, 48H, and 96H. In addition, GM‐CSF concentrations at 5H were significantly greater than 48H and 96H. No significant differences were noted between the groups in the GM‐CSF AUC response (*F* = 0.315, *P *=* *0.732).

No significant interactions (*F* = 1.297, *P *=* *0.250, *η* ^2^ = 0.071) were observed for changes in circulating IL‐8 concentrations. In addition, no differences were noted in the AUC response (*F* = 0.147, *P *=* *0.859) for circulating IL‐8 concentration.

### Intramuscular cytokine protein content

No significant interaction was observed for intramuscular content of G‐CSF or GM‐CSF (*F* = 0.222, *P *=* *0.937, *η* ^2^ = 0.018 and *F* = 0.345, *P *=* *0.911, *η* ^2^ = 0.027, respectively). Additionally, no differences were observed between groups for the AUC analysis of intramuscular G‐CSF or GM‐CSF contents (*F* = 0.042, *P *=* *0.959 and *F* = 2.109, *P *=* *0.142, respectively).

**Figure 3:**
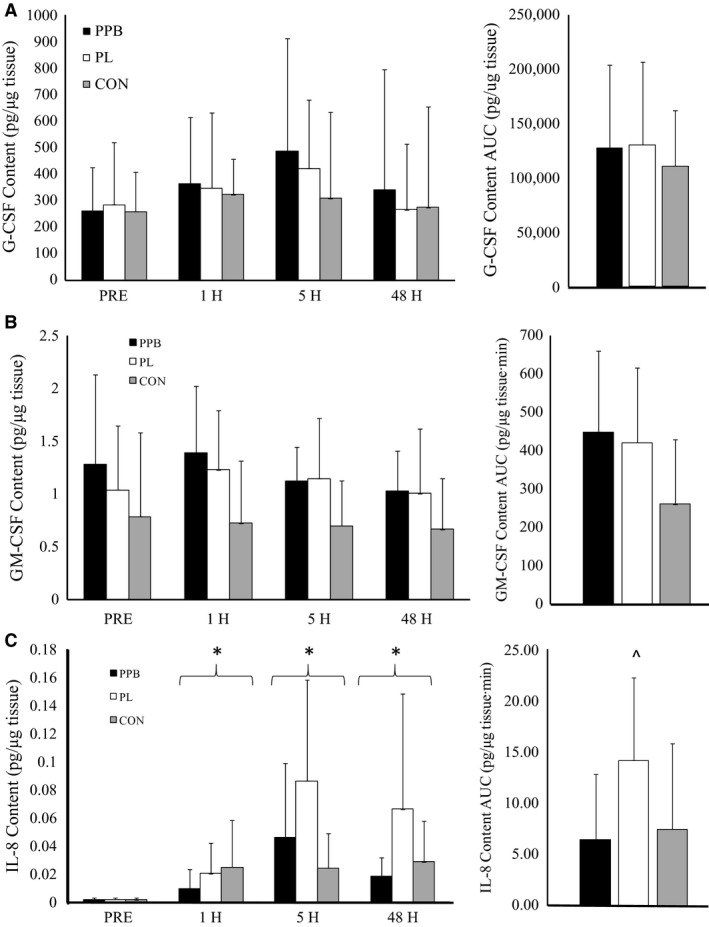
Intramuscular protein content following resistance exercise. Intramuscular protein content of (A) granulocyte colony stimulating factor (G‐CSF), (B) granulocyte–macrophage colony stimulating factor (GM‐CSF), and (C) interleukin‐8 (IL‐8). Supplement (PPB), placebo (PL), and control (CON) were analyzed for intramuscular protein content prior to exercise (PRE) as well as 1 h (1H), 5 h (5H), and 48 h (48H) post exercise. *Significantly different than PRE (*P *<* *0.05). ^^^Significantly different than PPB (*P *<* *0.05). Data presented as mean ± SD.

Intramuscular IL‐8 protein content is depicted in Figure 3. No significant interaction was observed for intramuscular IL‐8 content (*F* = 1.027, *P *=* *0.408, *η* ^2^ = 0.079), however, a significant time (*F* = 48.866, *P *<* *0.001, *η* ^2^ = 0.671) and group (*F* = 4.740, *P *=* *0.018, *η* ^2^ = 0.283) effect was observed. When groups were collapsed, pairwise comparisons indicated that skeletal muscle content of IL‐8 was significantly increased compared to PRE at 1H (*P *<* *0.001), 5H (*P *<* *0.001), and 48H (*P *<* *0.001). Additionally, when collapsed across time, pairwise comparisons indicated PL was significantly greater than CON (*P *=* *0.024) and PPB (*P *=* *0.010). Furthermore, AUC analysis indicated a significant difference between groups (F = 4.090; *P *=* *0.030). Post hoc analysis indicated PL was significantly greater than PPB (*P *=* *0.011), while a trend toward a difference was observed between PL and CON (*P *=* *0.066).

### Granulocyte percentage

**Figure 4:**
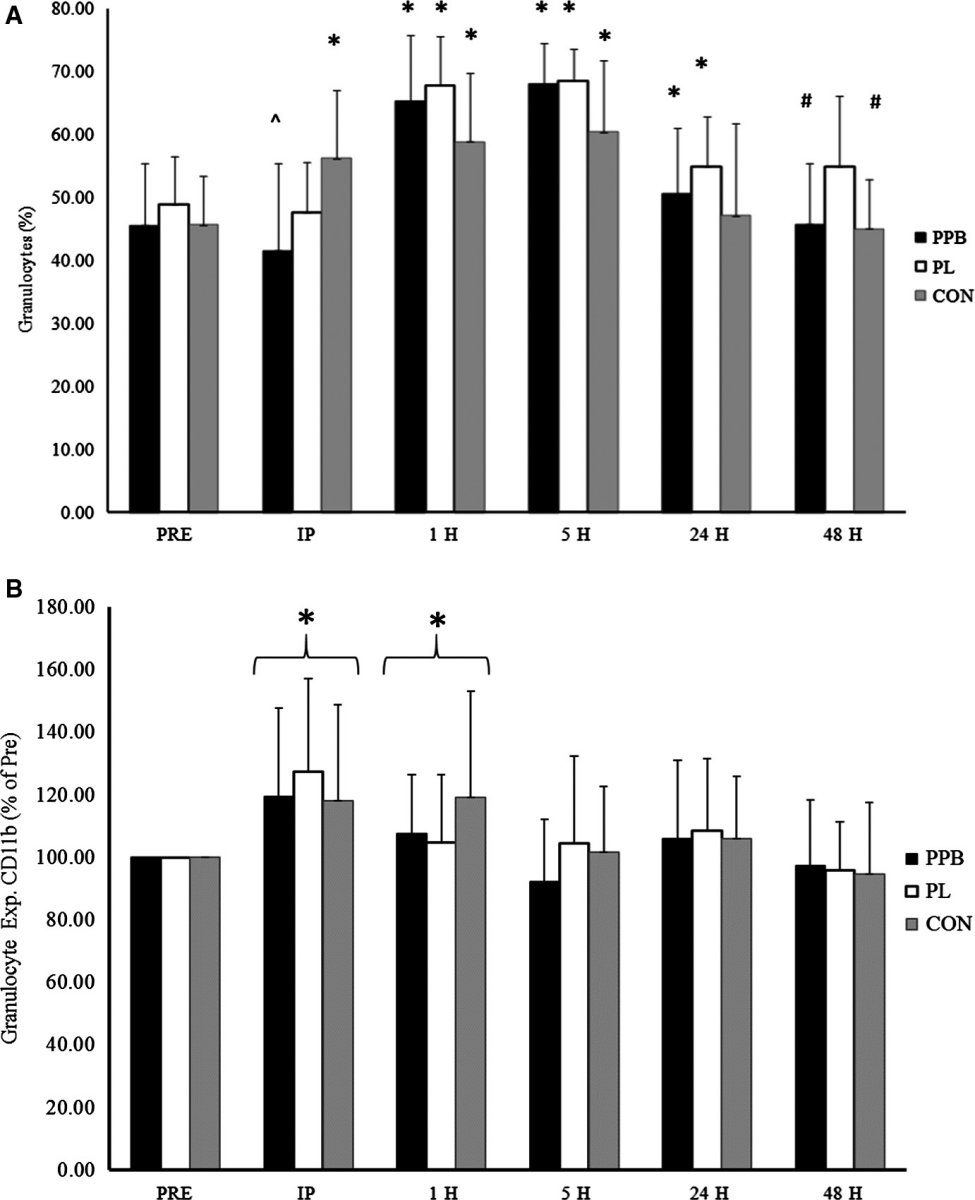
Granulocyte characteristics following resistance exercise. Circulating granulocyte (A) proportions and (B) adhesion characteristics. Supplement (PPB), placebo (PL), and control (CON) groups were analyzed for changes in the granulocyte characteristics pre (PRE) as well as immediately (IP), 1 h (1H), 5 h (5H), 24 h (24H), and 48 h (48H) post exercise. *Significantly different than corresponding value for PRE (*P *<* *0.05). ^^^Significantly different than corresponding value for CON (*P *<* *0.05). ^#^Significantly different than corresponding value for PL (*P *<* *0.05). Data presented as mean ± SD.

Changes in the granulocyte percentage in circulation in response to resistance exercise are depicted in Figure 4A. A significant interaction was observed in the percentage of circulating granulocytes (*F* = 5.150, *P *<* *0.001, *η* ^2^ = 0.233). Granulocyte percentage at IP were significantly higher in CON compared to PPB (*P *=* *0.004). Furthermore, a trend toward a difference was noted at IP between CON and PL (*P *=* *0.073). Additionally, granulocyte percentage in CON at 5H was significantly lower compared to PPB (*P *=* *0.022) and PL (*P *=* *0.013). Granulocyte percentage for PL was significantly greater at 48H than CON (*P *=* *0.026) or PPB (*P *=* *0.022).

### Granulocyte adhesion

Significant differences were observed between groups at PRE for granulocyte CD11b expression (*F* = 5.334, *P *=* *0.010). As such, analyses between groups for granulocyte CD11b expression were analyzed as the percent of resting values (PRE set at 100%).

Changes in granulocyte CD11b expression are depicted in Figure 4B. When analyzed as a percent of the resting values, no significant interaction was observed for the percent change in granulocyte CD11b expression (*F* = 1.022, *P *=* *0.421, *η* ^2^ = 0.057), however, a significant main effect for time was observed (*F* = 12.059, *P *<* *0.001, *η* ^2^ = 0.262). When collapsed across groups, pairwise comparisons indicated that the change in granulocyte CD11b expression from PRE to IP (121.6 ± 30.1%; *P *<* *0.001) and 1H (110.5 ± 24.9%; *P *=* *0.015) were significant, while a trend for a significant change in granulocyte CD11b expression from PRE to 24H (106.9 ± 23.6%; *P *=* *0.086) was also observed. Additionally, the change from PRE to IP was significantly greater than the change from PRE to 1H (*P *=* *0.001), 5H (99.5 ± 24.3%; *P *<* *0.001), 24H (*P *=* *0.001), and 48H (96.1 ± 19.9%; *P *<* *0.001). Furthermore, the change in CD11b expression on granulocytes from PRE to 1H was significantly greater than the change from PRE to 5H (*P *=* *0.016) and 48H (*P *=* *0.001).

### Correlations

Significant correlations were observed between markers of muscle damage and granulocyte percentage as well as changes in CD11b expression on granulocytes. Myoglobin AUC was significantly correlated with the percentage of granulocytes at IP (*r *=* *−0.515, *P *=* *0.001), 1H (*r *=* *−0.374, *P *=* *0.025), and 48H (*r *=* *−0.408, *P *=* *0.013), while the percentage of granulocytes at IP significantly correlated with CK concentrations at 24H (*r *=* *−0.635, *P *<* *0.001) and 48H (*r *=* *−0.565, *P *<* *0.001). CK concentrations at 48H were also significantly correlated with the change in granulocyte CD11b expression at IP on granulocytes (*r *=* *0.340, *P *=* *0.045). No significant correlations were observed between circulating and intramuscular cytokine concentrations.

## Discussion

Resistance exercise resulted in significant muscle damage in both PPB and PL, however, the magnitude of muscle damage appeared to be greater in PPB than PL, as reflected by differences in CK concentrations. Circulating IL‐8 was unchanged in response to resistance exercise, while G‐CSF and GM‐CSF increased independent of group. Intramuscular protein content of IL‐8 was significantly elevated following exercise, and was greater in PL than PPB, and trended toward a difference between PL and CON (*P *=* *0.066). No differences were observed for intramuscular protein content of G‐CSF or GM‐CSF in response to exercise or supplementation. Granulocyte percentage was significantly elevated in CON from IP to 5H postresistance exercise, while increases in granulocyte percentage in PPB and PL were delayed (1H–24H). Change in CD11b/CD18 expression occurred regardless of group, and was elevated on granulocytes immediately following resistance exercise at 1H. Markers of muscle damage were moderately correlated to granulocyte proportions and CD11b/CD18 expression following exercise (*r *=* *0.340–0.635), while no relationships were observed between circulating and intramuscular cytokines.

The primary function of G‐CSF and GM‐CSF is to stimulate granulocyte production and activation from the bone marrow (Roberts 2005; Francisco‐Cruz et al. 2014), thus the increase in the circulating concentration of these specific cytokines were consistent with their physiological roles. One investigation has previously demonstrated an increase in G‐CSF following dynamic resistance exercise (Mooren et al. 2012). To our knowledge, however, this is the first investigation to examine the response of GM‐CSF to a dynamic resistance exercise bout. Examination of the GM‐CSF response to exercise designed to elicit muscle damage has been minimal, with no changes previously reported (Smith et al. 2000). G‐CSF, however, has been demonstrated to increase (Paulsen et al. 2005; Wright et al. 2015), and remain unchanged (Smith et al. 2000; Ross et al. 2010) following exercise‐induced muscle damage. Exercise volume appears to drive this response as exercise protocols with a greater volume (300 eccentric contractions of knee extensors) being reported to elicit an increase in circulating G‐CSF (Paulsen et al. 2005; Wright et al. 2015), while lower training volumes (~50 eccentric contractions of knee flexors or hip extensors) did not (Smith et al. 2000; Ross et al. 2010).

In vitro analysis has suggested that skeletal muscle produces G‐CSF and GM‐CSF in a dose dependent fashion following mechanical stretch injury (Peterson and Pizza 2009). To our knowledge, no investigations have examined GM‐CSF protein content in skeletal muscle following exercise in vivo. Wright et al. (2015) demonstrated no change in protein content of G‐CSF following exercise‐induced muscle damage. The findings of Wright et al. (2015) are consistent with our findings, and may suggest that resistance exercise does not elicit production of G‐CSF from skeletal muscle. Rather, production of circulating G‐CSF may be a function of systemic inflammation associated with resistance exercise (Roberts 2005; Wright et al. 2015). Regardless of production site, the primary action of G‐CSF and GM‐CSF is the production and activation of granulocytes from the bone marrow (Roberts 2005; Francisco‐Cruz et al. 2014). Therefore, circulating concentrations may be more indicative of the overall function of these cytokines.

While GM‐CSF has been implicated in chemotaxis of granulocytes, IL‐8 also serves as a potent chemoattractant (Ribeiro et al. 1991; Peterson and Pizza 2009). We observed no change in circulating IL‐8 concentrations in response to resistance exercise. This is in contrast with Nieman et al. (2004), who observed significant increases immediately and 1 h following resistance exercise, but is supported by Buford et al. (2009). Training status does not appear to have influenced these results, as most investigations utilized trained participants (Nieman et al. 2004; Buford et al. 2009; Ross et al. 2010). However, the volume of exercise used in this study (140 total repetitions) was less than that of Nieman et al. (2004) (400 total repetitions; 4 sets of 10 repetitions in 10 different exercises), and may not have been sufficient to elicit a significant increase in circulating IL‐8 concentrations.

Due to the function of IL‐8 as a chemoattractant for neutrophils (Ribeiro et al. 1991), the lack of change in circulating IL‐8 is unclear. Previous research, however, has consistently demonstrated increased mRNA expression (Nieman et al. 2004; Buford et al. 2009; Della Gatta et al. 2014) and protein content (Della Gatta et al. 2014) of IL‐8 within the skeletal muscle following resistance exercise. Our results indicated a significant elevation in intramuscular IL‐8 protein content following resistance exercise, while AUC analysis revealed a reduced exercise response following polyphenol supplementation. As the elevations observed in the control group in this study contrast with that of Della Gatta et al. (2014), IL‐8 protein content was greater in PL than PPB and CON. Furthermore, AUC analysis does indicate a trend toward a greater response of IL‐8 to resistance exercise in PL compared to CON (*P *=* *0.066). Therefore, our results appear to support the findings of Della Gatta et al. (2014), while also indicating polyphenol supplementation may reduce the IL‐8 response to resistance exercise.

Previous investigations have demonstrated no impact of polyphenol supplementation in conjunction with exercise on the circulating concentration of IL‐8 (Nieman et al. 2013, 2007). To our knowledge, however, no investigations have examined the impact of polyphenols on IL‐8 within skeletal muscle nor other tissue in vivo. Evidence from in vitro models have demonstrated reduced IL‐8 production in various tissues following polyphenol administration (Chen et al. 2002; Trompezinski et al. 2003), as well as reduced chemotaxis of neutrophils (Takano et al. 2004). Therefore, the reduced IL‐8 response in skeletal muscle following resistance exercise may represent a reduced proinflammatory response that is promoted by polyphenol supplementation following acute resistance exercise.

In this study, increases in circulating G‐CSF and GM‐CSF appeared to precede the granulocytosis observed at 1H, 5H, and 24H following exercise. Expansion of the neutrophil population has been previously demonstrated in conjunction with increased circulating G‐CSF (Paulsen et al. 2005). Selective expansion of the granulocyte population is also well documented following resistance exercise (Miles et al. 1985; Ramel et al. 2003; Nieman et al. 2004), and exercise designed to elicit muscle damage (Paulsen et al. 2005). Evidence from an investigation designed to elicit muscle damage demonstrated that neutrophils return to baseline concentrations 24 h after exercise (Paulsen et al. 2005). Investigations examining dynamic resistance exercise, however, typically do not report the overall granulocyte population following 24 h of recovery in healthy participants (Miles et al. 1985; Ramel et al. 2003; Nieman et al. 2004). It has been previously suggested that granulocytes increase immediately following resistance exercise for approximately 2 h (Freidenreich and Volek 2012). Interestingly, this investigation observed increases in the granulocyte percentage at 1H following exercise, while CON observed increases at IP. Malm et al. (2000) previously reported significant increases in the neutrophil percentage immediately following muscle biopsies, with decreases in the lymphocyte percentage. Immediately following exercise, however, the neutrophil and lymphocyte percentages were maintained (Malm et al. 2000). Therefore, it appears that other cell types may increase following exercise and may have artificially masked the absolute increase in the granulocytes following exercise within this study. Therefore, the results of this investigation appear to indicate that the granulocyte expansion may be consistent with previous research, and may be prolonged (up to 24 h) following intense resistance exercise. Further investigation appears to be warranted.

In response to polyphenol supplementation, we observed a significantly greater circulating granulocyte percentage in PL than PPB and CON at 48H. Kerksick et al. (2010) have previously demonstrated a significant decrease in neutrophil concentration from 6H posteccentric exercise to 48H in participants supplementing with EGCG, while the PL group did not decrease. Given the chemoattractant properties of IL‐8 for neutrophils (Ribeiro et al. 1991), the reduced granulocyte proportion observed in this investigation may be related to the reduced intramuscular IL‐8 as opposed to circulating IL‐8. These findings may implicate polyphenol supplementation as a means to reduce the proinflammatory response following resistance exercise.

The coupling of CD11b with CD18 to form CD11b/CD18, and its involvement within the transendothelial migration process (Tan 2012), makes CD11b a key regulator of phagocyte migration to damaged tissue (Ley et al. 2007). Granulocytes migrate to damaged tissue in the initial hours following exercise (Paulsen et al. 2010). The increased CD11b/CD18 expression on granulocytes at IP and 1H observed in this study is consistent with the temporal appearance of these cells in damaged tissue. Currently, little consensus as to the time course of the granulocyte CD11b expression has been accepted following exercise (Peake et al. 2005a).

Investigations utilizing aerobic exercise (van Eeden et al. 1999; Jordan et al. 1999) and exercise designed to elicit muscle damage (Pizza et al. 1996; Saxton et al. 2003; Peake et al. 2005b) have previously examined CD11b expression on granulocytes. However, no investigations that we are aware of have examined the impact of dynamic resistance exercise on CD11b expression on granulocytes. Previously, Malm et al. (2000) has demonstrated muscle biopsies to impact the immune response. Our results indicate that muscle biopsies can stimulate increases in CD11b expression on granulocytes at IP and 1H; however, it is difficult to distinguish between the effect of the muscle biopsy and exercise in this study. Evidence from previous investigations indicates exercise may also influence the CD11b response on granulocytes. Various studies have reported significant increases immediately following aerobic exercise (van Eeden et al. 1999; Jordan et al. 1999), which may persist for up to 30 min postexercise (van Eeden et al. 1999). Following downhill running, however, Peake et al. (2005b) reported no changes in CD11b expression during the recovery period in well‐trained men. Similarly, studies examining 50 eccentric contractions of the quadriceps (Saxton et al. 2003) or 25 eccentric contractions of the elbow flexors (Pizza et al. 1996) in moderately trained men, reported no change in CD11b expression up to 24 h postexercise. The studies evaluating CD11b post downhill run and eccentric contractions, however, first measured CD11b expression at 4 and 1.5 h postexercise, respectively. We observed an increase in the expression of CD11b on granulocytes at IP and 1H, which was prior to any of the measurements assessed by Saxton et al. (2003) or Pizza et al. (1996). Pizza et al. (1996) did demonstrate a significant increase in CD11b expression 24 h following their damaging protocol. The results of this present study provide some support as we indicated a trend for an increased expression at 24H (*P *=* *0.086).

Taken together, the present study demonstrates that an acute, intense resistance exercise bout will stimulate a proinflammatory response of the immune system in untrained men. Furthermore, polyphenol supplementation may limit this proinflammatory response, specifically by reducing intramuscular IL‐8 protein content. As shown in previous research, it does appear that muscle biopsies may exacerbate this immune response (Malm et al. 2000). Since the current study design necessitated the use of biopsies for assessment of intramuscular cytokine content, future research should aim to confirm the findings of this study in circulation without the potential influence of skeletal muscle biopsies. Furthermore, as absolute immune cell counts were not obtained, the findings of this study are limited to the proportion of granulocytes within circulation, relative to the total number of leukocytes. Future research should focus on the absolute changes of these populations as well as the impact of polyphenol supplementation on other aspects of the immune response to resistance exercise.

## Conflict of Interest

Kelli A. Herrlinger is an employee of Kemin Foods, L.C. All other authors have no actual or potential conflicts of interest to report.

## References

[phy213058-bib-0001] Brzycki, M. 1993 Strength testing ‐ predicting a one‐rep max from reps‐to‐fatigue. J. Phy. Edu. Recr. Dance 64:88–90.

[phy213058-bib-0002] Buford, T. W. , M. B. Cooke , and D. S. Willoughby . 2009 Resistance exercise‐induced changes of inflammatory gene expression within human skeletal muscle. Eur. J. Appl. Physiol. 107:463–471. 19669788 10.1007/s00421-009-1145-z

[phy213058-bib-0003] Chen, P. C. , D. S. Wheeler , V. Malhotra , K. Odoms , A. G. Denenberg , and H. R. Wong . 2002 A green tea‐derived polyphenol, epigallocatechin‐3‐gallate, inhibits IkappaB kinase activation and IL‐8 gene expression in respiratory epithelium. Inflammation 26:233–241. 12238566 10.1023/A:1019718718977 PMC7101574

[phy213058-bib-0004] Clarkson, P. M. , and M. J. Hubal . 2002 Exercise‐induced muscle damage in humans. Am. J. Phys. Med. Rehabil. 81(11 Suppl):S52–S69. 12409811 10.1097/00002060-200211001-00007

[phy213058-bib-0005] Della Gatta, P. A. **,** D. Cameron‐Smith , and J. M. Peake . 2014 Acute resistance exercise increases the expression of chemotactic factors within skeletal muscle. Eur. J. Appl. Physiol. 114:2157–2167. 24968868 10.1007/s00421-014-2936-4

[phy213058-bib-0006] Dill, D. B. , and D. L. Costill . 1974 Calculation of percentage changes in volumes of blood, plasma, and red cells in dehydration. J. Appl. Physiol. 37:247–248. 4850854 10.1152/jappl.1974.37.2.247

[phy213058-bib-0007] van Eeden, S. F. , J. Granton , J. M. Hards , B. Moore , and J. C. Hogg . 1999 Expression of the cell adhesion molecules on leukocytes that demarginate during acute maximal exercise. J. Appl. Physiol. 86:970–976. 10066712 10.1152/jappl.1999.86.3.970

[phy213058-bib-0008] Francisco‐Cruz, A. , M. Aguilar‐Santelises , O. Ramos‐Espinosa , et al. 2014 Granulocyte‐macrophage colony‐stimulating factor: not just another haematopoietic growth factor. Med. Oncol. 31:774. 24264600 10.1007/s12032-013-0774-6

[phy213058-bib-0009] Freidenreich, D. J. , and J. S. Volek . 2012 Immune responses to resistance exercise. Exer. Immunol. Rev. 18:8–41. 22876721

[phy213058-bib-0010] Hammond, M. E. , G. R. Lapointe , P. H. Feucht , et al. 1995 IL‐8 induces neutrophil chemotaxis predominantly via type I IL‐8 receptors. J Immunol. 155:1428–1433. 7636208

[phy213058-bib-0011] Herrlinger, K. A. , D. M. Chirouzes , and M. A. Ceddia . 2015 Supplementation with a polyphenolic blend improves post‐exercise strength recovery and muscle soreness. Food Nutr. Res. 59:30034. 26689317 10.3402/fnr.v59.30034 PMC4685974

[phy213058-bib-0012] Hoffman, J. R. 2006 Pp. 32–34 Norms for fitness, performance, and health. Human Kinetics, Champaign (IL).

[phy213058-bib-0013] Jordan, J. , R. Beneke , M. Hutler , A. Veith , F. C. Luft , and H. Haller . 1999 Regulation of MAC‐1 (CD11b/CD18) expression on circulating granulocytes in endurance runners. Med. Sci. Sports Exerc. 31:362–367. 10188738 10.1097/00005768-199903000-00002

[phy213058-bib-0014] Jowko, E. , J. Sacharuk , B. Balasinska , P. Ostaszewski , M. Charmas , and R. Charmas . 2011 Green tea extract supplementation gives protection against exercise‐induced oxidative damage in healthy men. Nutr. Res. 31:813–821. 22118751 10.1016/j.nutres.2011.09.020

[phy213058-bib-0015] Kawai, K. , N. H. Tsuno , J. Kitayama , et al. 2004 Epigallocatechin gallate attenuates adhesion and migration of CD8+ T cells by binding to CD11b. J. Allergy Clin. Immunol. 113:1211–1217. 15208607 10.1016/j.jaci.2004.02.044

[phy213058-bib-0016] Kerksick, C. M. , R. B. Kreider , and D. S. Willoughby . 2010 Intramuscular adaptations to eccentric exercise and antioxidant supplementation. Amino Acids 39:219–232. 19967420 10.1007/s00726-009-0432-7

[phy213058-bib-0017] Ley, K. , C. Laudanna , M. I. Cybulsky , and S. Nourshargh . 2007 Getting to the site of inflammation: the leukocyte adhesion cascade updated. Nat. Rev. Immunol. 7:678–689. 17717539 10.1038/nri2156

[phy213058-bib-0018] Malm, C. , P. Nyberg , M. Engstrom , et al. 2000 Immunological changes in human skeletal muscle and blood after eccentric exercise and multiple biopsies. J. Physiol. 529:243–262. 11080266 10.1111/j.1469-7793.2000.00243.x PMC2270185

[phy213058-bib-0019] Merry, T. L. , and M. Ristow . 2015 Do antioxidant supplements interfere with skeletal muscle adaptation to exercise training? J. Physiol. 594:5135–5147. 10.1113/JP270654 PMC5023714 26638792

[phy213058-bib-0020] Miles, M. P. , S. K. Leach , W. J. Kraemer , K. Dohi , J. A. Bush , and A. M. Mastro . 1985 Leukocyte adhesion molecule expression during intense resistance exercise. J. Appl. Physiol. 1998:1604–1609. 10.1152/jappl.1998.84.5.1604 9572805

[phy213058-bib-0021] Mooren, F. C. , K. Volker , R. Klocke , S. Nikol , J. Waltenberger , and K. Kruger . 2012 Exercise delays neutrophil apoptosis by a G‐CSF‐dependent mechanism. J. Appl. Physiol. 113:1082–1090. 22858628 10.1152/japplphysiol.00797.2012

[phy213058-bib-0022] Nguyen, H. X. , and J. G. Tidball . 2003 Null mutation of gp91phox reduces muscle membrane lysis during muscle inflammation in mice. J. Physiol. 553(Pt 3):833–841. 14555723 10.1113/jphysiol.2003.051912 PMC2343638

[phy213058-bib-0023] Nieman, D. C. , J. M. Davis , V. A. Brown , et al. 2004 Influence of carbohydrate ingestion on immune changes after 2 h of intensive resistance training. J. Appl. Physiol. 96:1292–1298. 14672962 10.1152/japplphysiol.01064.2003

[phy213058-bib-0024] Nieman, D. C. , D. A. Henson , J. M. Davis , et al. 2007 Quercetin ingestion does not alter cytokine changes in athletes competing in the Western States Endurance Run. J. Interferon Cytokine Res. 27:1003–1011. 18184041 10.1089/jir.2007.0050

[phy213058-bib-0025] Nieman, D. C. , N. D. Gillitt , A. M. Knab , et al. 2013 Influence of a polyphenol‐enriched protein powder on exercise‐induced inflammation and oxidative stress in athletes: a randomized trial using a metabolomics approach. PLoS ONE 8:e72215. 23967286 10.1371/journal.pone.0072215 PMC3744465

[phy213058-bib-0026] O'Fallon, K. S. , D. Kaushik , B. Michniak‐Kohn , C. P. Dunne , E. J. Zambraski , and P. M. Clarkson . 2012 Effects of quercetin supplementation on markers of muscle damage and inflammation after eccentric exercise. Int J Sport Nutr Exerc. Metab. 22:430–437. 22805422 10.1123/ijsnem.22.6.430

[phy213058-bib-0027] Panza, V. S. , E. Wazlawik , G. Ricardo Schutz , L. Comin , K. C. Hecht , E. L. da Silva . 2008 Consumption of green tea favorably affects oxidative stress markers in weight‐trained men. Nutrition 24:433–442. 18337059 10.1016/j.nut.2008.01.009

[phy213058-bib-0028] Parkin, J. , and B. Cohen . 2001 An overview of the immune system. Lancet 357:1777–1789. 11403834 10.1016/S0140-6736(00)04904-7

[phy213058-bib-0029] Paulsen, G. , H. B. Benestad , I. Strom‐Gundersen , L. Morkrid , K. T. Lappegard , and T. Raastad . 2005 Delayed leukocytosis and cytokine response to high‐force eccentric exercise. Med. Sci. Sports Exerc. 37:1877–1883. 16286856 10.1249/01.mss.0000177064.65927.98

[phy213058-bib-0030] Paulsen, G. , R. Crameri , H. B. Benestad , et al. 2010 Time course of leukocyte accumulation in human muscle after eccentric exercise. Med. Sci. Sports Exerc. 42:75–85. 20010127 10.1249/MSS.0b013e3181ac7adb

[phy213058-bib-0031] Paulsen, G. , H. Hamarsland , K. T. Cumming , et al. 2014 Vitamin C and E supplementation alters protein signalling after a strength training session, but not muscle growth during 10 weeks of training. J. Physiol. 592:5391–5408. 25384788 10.1113/jphysiol.2014.279950 PMC4270502

[phy213058-bib-0032] Peake, J. , K. Nosaka , and K. Suzuki . 2005a Characterization of inflammatory responses to eccentric exercise in humans. Exer. Immunol. Rev. 11:64–85. 16385845

[phy213058-bib-0033] Peake, J. M. , K. Suzuki , G. Wilson , et al. 2005b Exercise‐induced muscle damage, plasma cytokines, and markers of neutrophil activation. Med. Sci. Sports Exerc. 37:737–745. 15870626 10.1249/01.mss.0000161804.05399.3b

[phy213058-bib-0034] Peterson, J. M. , and F. X. Pizza . 2009 Cytokines derived from cultured skeletal muscle cells after mechanical strain promote neutrophil chemotaxis in vitro. J. Appl. Physiol. 1985 106:130–137. 18974369 10.1152/japplphysiol.90584.2008

[phy213058-bib-0035] Pizza, F. X. , B. H. Davis , S. D. Henrickson , et al. 1996 Adaptation to eccentric exercise: effect on CD64 and CD11b/CD18 expression. J. Appl. Physiol. 80:47–55. 8847330 10.1152/jappl.1996.80.1.47

[phy213058-bib-0036] Pizza, F. X. , J. M. Peterson , J. H. Baas , and T. J. Koh . 2005 Neutrophils contribute to muscle injury and impair its resolution after lengthening contractions in mice. J. Physiol. 562(Pt 3):899–913. 15550464 10.1113/jphysiol.2004.073965 PMC1665528

[phy213058-bib-0037] Ramel, A. , K. H. Wagner , and I. Elmadfa . 2003 Acute impact of submaximal resistance exercise on immunological and hormonal parameters in young men. J. Sports Sci. 21:1001–1008. 14748457 10.1080/02640410310001641395

[phy213058-bib-0038] Ribeiro, R. A. , C. A. Flores , F. Q. Cunha , and S. H. Ferreira . 1991 IL‐8 causes in vivo neutrophil migration by a cell‐dependent mechanism. Immunology 73:472–477. 1916898 PMC1384579

[phy213058-bib-0039] Roberts, A. W. 2005 G‐CSF: a key regulator of neutrophil production, but that's not all!. Growth Factors 23:33–41. 16019425 10.1080/08977190500055836

[phy213058-bib-0040] Ross, M. L. , S. L. Halson , K. Suzuki , et al. 2010 Cytokine responses to carbohydrate ingestion during recovery from exercise‐induced muscle injury. J. Interferon Cytokine Res. 30:329–337. 20187772 10.1089/jir.2009.0079

[phy213058-bib-0041] Saxton, J. M. , D. Claxton , E. Winter , and A. G. Pockley . 2003 Peripheral blood leucocyte functional responses to acute eccentric exercise in humans are influenced by systemic stress, but not by exercise‐induced muscle damage. Clin. Sci. (Lond.) 104:69–77. 12519089 10.1042/

[phy213058-bib-0042] Smith, L. L. , A. Anwar , M. Fragen , C. Rananto , R. Johnson , and D. Holbert . 2000 Cytokines and cell adhesion molecules associated with high‐intensity eccentric exercise. Eur. J. Appl. Physiol. 82(1–2):61–67. 10879444 10.1007/s004210050652

[phy213058-bib-0043] Takano, K. , K. Nakaima , M. Nitta , F. Shibata , and H. Nakagawa . 2004 Inhibitory effect of (‐)‐epigallocatechin 3‐gallate, a polyphenol of green tea, on neutrophil chemotaxis in vitro and in vivo. J. Agric. Food Chem. 52:4571–4576. 15237969 10.1021/jf0355194

[phy213058-bib-0044] Tan, S. M. 2012 The leucocyte beta2 (CD18) integrins: the structure, functional regulation and signalling properties. Biosci. Rep. 32:241–269. 22458844 10.1042/BSR20110101

[phy213058-bib-0045] Tidball, J. G. , and S. A. Villalta . 2010 Regulatory interactions between muscle and the immune system during muscle regeneration. Am. J. Physiol. Regul. Integr. Comp. Physiol. 298:R1173–R1187. 20219869 10.1152/ajpregu.00735.2009 PMC2867520

[phy213058-bib-0046] Townsend, J. R. , J. R. Hoffman , M. S. Fragala , et al. 2016 A microbiopsy method for immunohistological and morphological analysis: a pilot study. Med. Sci. Sports Exerc. 48:331–335. 10.1249/MSS.0000000000000772 26375254

[phy213058-bib-0047] Trompezinski, S. , A. Denis , D. Schmitt , and J. Viac . 2003 Comparative effects of polyphenols from green tea (EGCG) and soybean (genistein) on VEGF and IL‐8 release from normal human keratinocytes stimulated with the proinflammatory cytokine TNFalpha. Arch. Dermatol. Res. 295:112–116. 12811578 10.1007/s00403-003-0402-y

[phy213058-bib-0048] Vassilakopoulos, T. , M. H. Karatza , P. Katsaounou , A. Kollintza , S. Zakynthinos , and C. Roussos . 2003 Antioxidants attenuate the plasma cytokine response to exercise in humans. J. Appl. Physiol. 1985 94:1025–1032. 12571133 10.1152/japplphysiol.00735.2002

[phy213058-bib-0049] Wright, C. R. , E. L. Brown , P. A. Della Gatta , et al. 2015 Regulation of granulocyte colony‐stimulating factor and its receptor in skeletal muscle is dependent upon the type of inflammatory stimulus. J. Interferon Cytokine Res. 35:710–719. 26057332 10.1089/jir.2014.0159

